# The virtual-hand illusion: effects of impact and threat on perceived ownership and affective resonance

**DOI:** 10.3389/fpsyg.2013.00604

**Published:** 2013-09-06

**Authors:** Ke Ma, Bernhard Hommel

**Affiliations:** Institute for Psychological Research and Leiden Institute for Brain and Cognition, Leiden UniversityLeiden, Netherlands

**Keywords:** vibrotactile stimulation, rubber hand illusion, virtual hand illusion, body ownership, body awareness, threat, affective responses

## Abstract

The rubber hand illusion refers to the observation that participants perceive “body ownership” for a rubber hand if it moves, or is stroked in synchrony with the participant's real (covered) hand. Research indicates that events targeting artificial body parts can trigger affective responses (affective resonance) only with perceived body ownership, while neuroscientific findings suggest affective resonance irrespective of ownership (e.g., when observing other individuals under threat). We hypothesized that this may depend on the severity of the event. We first replicated previous findings that the rubber hand illusion can be extended to virtual hands—the virtual-hand illusion. We then tested whether hand ownership and affective resonance (assessed by galvanic skin conductance) are modulated by the experience of an event that either “impacted” (a ball hitting the hand) or “threatened” (a knife cutting the hand) the virtual hand. Ownership was stronger if the virtual hand moved synchronously with the participant's own hand, but this effect was independent from whether the hand was impacted or threatened. Affective resonance was mediated by ownership however: In the face of mere impact, participants showed more resonance in the synchronous condition (i.e., with perceived ownership) than in the asynchronous condition. In the face of threat, in turn, affective resonance was independent of synchronicity—participants were emotionally involved even if a threat was targeting a hand that they did not perceive as their own. Our findings suggest that perceived body ownership and affective responses to body-related impact or threat can be dissociated and are thus unlikely to represent the same underlying process. We argue that affective reactions to impact are produced in a top-down fashion if the impacted effector is assumed to be part of one's own body, whereas threatening events trigger affective responses more directly in a bottom-up fashion—irrespective of body ownership.

## Introduction

In the “rubber-hand illusion” (RHI) first reported by Botvinick and Cohen (1998), people feel that a rubber hand lying in front of them belongs to their own body if the rubber hand and their own unseen hand are being stroked synchronously. This observation has been replicated and extended in various studies. For example, Tsakiris and Haggard (2005) showed that, for the RHI to work, the rubber hand should look like and be aligned with one's own hand. Moreover, Armel and Ramachandran (2003) reported that the illusion goes along with elevated galvanic skin conductance responses (SCR) in the case of possible threat directed at the rubber hand, indicating a kind of “affective resonance” and “emotional involvement” with the artificial hand.

Recent research has provided evidence that the RHI can be induced through (sometimes immersive) virtual reality where the rubber hand is replaced by a virtual hand. A common method is to present participants with visual 3D images of the virtual hand on a screen in front of them, in some cases together with tactile stimulation of their real, hidden hand (Padilla et al., 2010). Sanchez-Vives et al. (2010) showed that a virtual hand illusion (VHI) can be induced even in the absence of tactile stimulation, simply by manipulating the temporal delay between the participant's own movement (as measured by a data glove) and the movements of the virtual hand on a screen. Slater et al. (2008) found reliable correlations between the impression of hand ownership and hand-related EMG activation, suggesting a connection between perceived ownership and action control.

Of particular interest for the present study, Yuan and Steed (2010) measured SCR responses to what they considered threats to a virtual hand and found similar elevations as with rubber hands. Participants were operating the hand of an avatar, which allowed them to play games in virtual space. At some point, a (virtual) lamp would fall on the virtual hand operated by the participant, which induced a reliable increase in SCR. In a control condition, the hand was replaced by an arrow, which produced significantly less increase in SCR. The SCR effects were mirrored by the effect obtained for the body-ownership questionnaire (Botvinick and Cohen, 1998): perceived ownership was significantly more pronounced in the hand condition than in the arrow condition. Taken together, these findings suggest that people emotionally “care” about what they perceive as being a part of their body but not, or not so much, about what they perceive as belonging to the body of someone else.

Even though this interpretation fits with previous observations from studies on the RHI, it does not seem to be fully consistent with research showing affective resonance when observing other people under threat or in pain. For instance, receiving a visual signal that a loved one will receive a painful electric shock has been found to activate the same brain areas (such as the dorsal anterior cingulate cortex and anterior insula) that are active when being in pain oneself (Singer et al., 2004). Even witnessing a stranger being treated with a painful pinprick stimulus activates the same areas that are active when receiving such a stimulus oneself (Morrison et al., 2004). Observations of that sort have been interpreted as indicating that people do not distinguish much between themselves and others if it comes to the representation of affect (Keysers, 2011), and the same argument has been made with respect to the actions (Gallese et al., 2004) and personalities (Hommel et al., 2009) of oneself and of others. This seems to imply that we care about others even when there is no body ownership, which does not seem to fit with Yuan and Steed's (2010) observation that people's affective response to the threat (as assessed by SCR) is much reduced in the absence of ownership. The aim of the present study was to resolve that issue, if possible.

The consideration of two aspects of Yuan and Steed's study might help explaining this seeming discrepancy. For one, they did not use the standard synchronization technique to induce different degrees of body ownership (such as inducing different temporal delay between movement of the actual hand and movement of the virtual hand); rather, they compared people's responses to what can be considered a plausible body part—a virtual hand—with responses to what can be considered an implausible body part—a visual arrow. Arguably, this might not only have induced the observed differences in perceived ownership but also prevented the cognitive representation of the arrow as a possible body part as such. It might thus be that people would care about a threatened virtual hand even if it would not be perceived as being a part of their own body—if it only is recognizable as a hand. In the present study, we tested this possibility by comparing people's perceived ownership and affective responses to virtual hands that they could operate with either no temporal delay (the synchronous condition) or with a considerable temporal delay (the asynchronous condition). Like in the study of Sanchez-Vives et al. (2010), we expected that perceived ownership would be significantly reduced in the asynchronous as compared to the synchronous condition. We also measured SCR to see whether and to what degree perceived ownership (i.e., synchronicity) would modulate the affective response to threats targeting the virtual hand.

For another, even though Yuan and Steed (2010) intended to investigate the affective response to threat, the threatening event merely consisted of a virtual lamp falling on the virtual hand. Even though the contact between the lamp and the hand was clearly visible to the participant, it is difficult to judge from the visual display how much pain, if any, this contact might have caused if it were real. Accordingly, the manipulation may have represented an “impact” of an object on the virtual hand rather than a degree of actual threat that would be comparable to an electric shock [as in Singer et al. (2004)] or a pinprick [as in Morrison et al. (2004)]. It is possible that some degree of severity of a threat is required to induce a high degree of cross-personal affective resonance as evidenced by the studies of Singer et al. and Morrison et al. To test this possibility, we combined the synchronicity manipulation with a manipulation of the object that would get in contact with the virtual hand. In one condition, the virtual hand was hit by a ball, which the participant could both see on a screen in front of her and feel in the palm of her hand. This impact was clearly noticeable but the speed of the ball was chosen in such a way that a real contact with the same parameters would not be perceived as painful. In another condition, the virtual hand was hit and actually cut by a knife—an event that was considered to represent a threat. Our main question was whether the synchronicity manipulation would affect the two conditions differently. In view of the various previous demonstrations of the VHI, we expected that the affective response to mere impact (the ball condition) should be more pronounced for the synchronous than for the asynchronous condition. However, more interesting was whether the synchronicity effect would be comparable with a real threat (the knife condition) or whether participants would show affective resonance to the threatened hand irrespective of hand ownership (i.e., of synchronicity).

Before conducting the actual experiment, we first investigated whether we could produce a reliable VHI with our equipment and which stimulus/feedback parameters would contribute to the illusion. In this pilot study, we presented participants with a virtual hand on a monitor in front of them. They were able to operate this virtual hand with their own, unseen hand by means of a data glove. In some trials, moving their own hand resulted in immediate movement of the virtual hand (synchronous condition) while in other trials the movements of the virtual hand were delayed (asynchronous condition). In some trials, participants only saw the movement of the virtual hand on the screen (visual conditions) while in other trials the virtual hand was hit by a ball, which was accompanied by a vibration in the palm of their own hand (visual-tactile conditions).

## Pilot study

### Methods

#### Participants

Twenty participants with mean age 22.2 ± 3.34 (SD) were recruited from Leiden University in exchange for course credit or pay. Informed consent was obtained from all participants before the experiment. Participants were naive with respect to the RHI/VHI. The study was approved by the Leiden University Human research ethics committee.

#### Experimental setup

**Figure 1:**
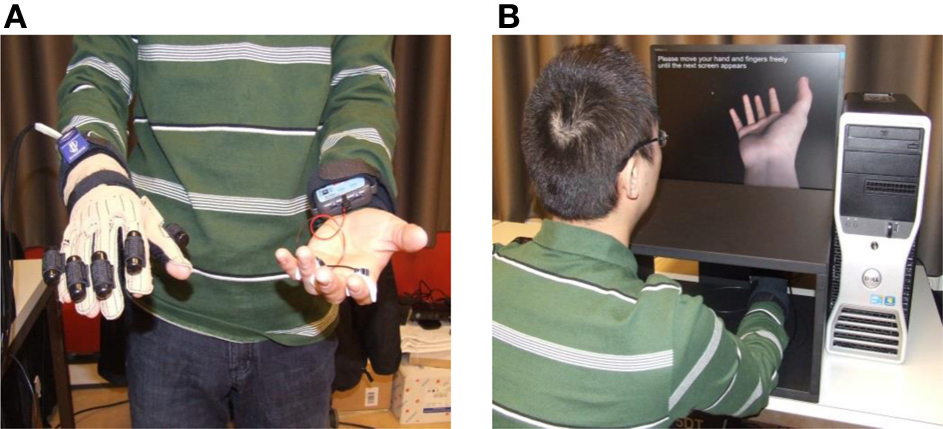
**The experimental setup. (A)** Participants wore a data glove with attached vibrators on their right hand. **(B)** Participants controlled a virtual hand on a screen by moving their real right hand.

The study was performed in a virtual reality environment. The setup consisted of a 3 degree-of-freedom orientation tracker (InterSense), a data glove (Cyberglove), and virtual reality software (Vizard). The Cyberglove has six vibration sensors attached, one on each finger and one on the palm; they are programmable to set the vibration time and strength. As shown in Figure 1A, participants wore the glove on their right hand and the InterSense tracker on their right wrist. We used a virtual hand from Vizard character set and imported the tracker and data glove module into Vizard, so that the virtual hand received the data from the tracker and data glove. We generated a virtual hand that was controlled by the participant's hand movement (see Figure 1B).

#### Design

There were two within-group factors: First, the movement of the virtual hand was either synchronous or asynchronous with the movement of the actual hand. Second, the virtual hand was either seen alone or hit by a virtual ball (as seen on the screen and felt in the palm of the hand). The order of the two synchronicity conditions was balanced across participants, as was the order of the visual and the visual-tactile conditions.

#### Procedure

Participants were seated in front of a computer monitor, wearing the glove on their right hand and the orientation tracker on their right wrist. At the beginning of each of the four trials, they were asked to move their fingers for 30 s in front of the black screen, which was necessary to initialize the system properly. Then the computer program generated a virtual hand on the screen and the trial started. In each of the four trials, participants were asked to move fingers and wrist for 1 min. In visual-tactile trials, they were asked to put their hand on the desk with the palm upwards, so that the contacting ball would hit the virtual hand at the palm. The ball bounced several times, every time associated with a vibration of the sensor located in the palm of the glove. In the synchronous trials, the virtual hand moved in synchrony with the participant's own hand and, in visual-tactile synchronous trials, the vibration was presented each time the ball contacted the hand. In the asynchronous trials, the movement of the virtual hand was delayed by 2 s and the vibration set in 2 s after each ball-hand contact. After the completion of each trial, participants worked through the questionnaire described below.

#### Questionnaire

To assess the extent to which participants experienced the VHI we used an adapted version of the standard nine-item questionnaire (Botvinick and Cohen, 1998; Slater et al., 2008; Padilla et al., 2010). For each statement, participants responded by choosing a score in a 5-point (0–4) Likert scale, ranging from 0 for “strongly disagree” to 4 for “strongly agree.” The statements were:

Q1. “Sometimes I had the sensation that vibration I felt on my hand was on the same location where the hand on the screen was in contact with the object,”

Q2. “Sometimes I had the sensation that the vibration I felt on my hand was caused by the contact of the object with the hand on the screen,”

Q3. “The movements of the hand on the screen were caused by myself,”

Q4. “It sometimes seemed my own hand was located on the screen,”

Q5. “The hand on the screen began to resemble my own hand, in terms of shape, skin tone, freckles, or some other usual feature,”

Q6. “Sometimes it seemed as if what I was feeling was caused by the ball that I was seeing on the screen,”

Q7. “Sometimes I felt as if the hand on the screen was my own hand,”

Q8. “Sometimes I felt as if my real hand was becoming virtual,”

Q9. “It seemed as if I might have more than one right hand”

Questions 1–4, 6, 7 are supposed to indicate the actual illusion, and Questions 5, 8, 9 are usually considered fillers. In the pilot, three more questions were included for explorative purposes, but they were unrelated to the illusion proper (“I felt that I can control the hand on the screen,” “sometimes I had the feeling that I was receiving the vibration in the location of the hand on the screen,” “sometimes it seems that the contact that I was feeling originated from the screen”) and were not further analyzed.

### Results

**Figure 2:**
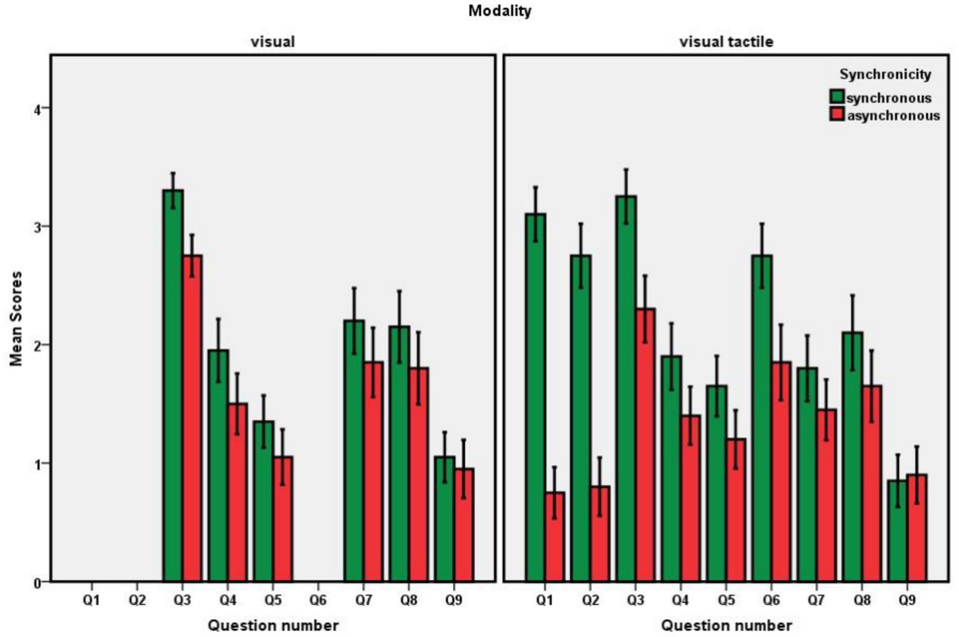
**Pilot study: Boxplots for the questionnaire scores as a function of modality and synchronicity**.

We analyzed the responses to items 1, 2, and 6 by means of a one-factorial (synchronicity) ANOVA and responses to the remaining items by means of a 2 (synchronicity) × 2 (modality) ANOVA. Because Questions 1, 2, and 6 were specific to the tactile modality, the boxplots in the left panel of Figure 2 only show the scores of the remaining questions; see Table 1 for means and standard deviation for all questions. For Questions 1–7 the analyses yielded reliable main effects of synchronicity (see Table 1 for *p*-values) but no other effects, all *p*s > 0.1. That is, all critical questions provided evidence for a VHI. This illusion was not reliably mediated by modality, but effects tended to be numerically larger for the visual-tactile condition.

**Table 1:** **Pilot study: Means and standard deviations (in brackets) for the questionnaire scores**.

	**Q1**	**Q2**	**Q3**	**Q4**	**Q5**	**Q6**	**Q7**	**Q8**	**Q9**
Visual synchronous			3.30 (0.657)	1.95 (1.191)	1.35 (0.988)		2.20 (1.240)	2.15 (1.348)	1.05 (0.945)
Visual asynchronous			2.75 (0.786)	1.50 (1.147)	1.05 (1.050)		1.85 (1.309)	1.80 (1.361)	0.95 (1.099)
Visual-tactile synchronous	3.10 (1.021)	2.75 (1.209)	3.25 (1.020)	1.90 (1.252)	1.65 (1.137)	2.75 (1.209)	1.80 (1.240)	2.10 (1.410)	0.85 (0.988)
Visual-tactile asynchronous	0.75 (0.967)	0.80 (1.105)	2.30 (1.261)	1.40 (1.095)	1.20 (1.105)	1.85 (1.424)	1.45 (1.146)	1.65 (1.348)	0.90 (1.071)
*p* (synchronicity)	<0.001	<0.001	0.003	0.031	0.004	0.014	0.049	0.107	0.867
*p* (modality)			0.196	0.702	0.154		0.111	0.585	0.234
*p* (interaction)			0.269	0.874	0.625		1.000	0.766	0.419

P-values for the main effects of synchronicity are shown in the last row.

### Discussion

The outcome is clear: we were able to replicate the VHI with our setup. The lack of an interaction with modality suggests that adding the tactile information is not required to generate reliable effects. Nevertheless, given that the numerical effects tended to be more pronounced for the conditions with tactile stimulation, we kept this setup for our experiment.

## Experiment

Our actual experiment made use of the visual-tactile stimulation condition, so that modality was no longer a factor. However, we introduced another factor: type of event. In all four conditions of the experiment, the hand was contacted by an object. In the “impact” condition, this was a ball hitting the virtual hand like in the visual-tactile conditions of the pilot study. In the “threat” condition, this was a knife cutting the virtual hand, which caused some blood appearing from the thus-created wound. To assess affective resonance, we included SCR as a second measure.

### Methods

#### Participants

18 participants with mean age 23.6 ± 4.7 (SD) were recruited from Leiden University in exchange for course credit or pay. Informed consent was obtained from all participants before the experiment. Participants were naive with respect to the RHI/VHI. The experiment was approved by the Leiden University Human research ethics committee.

#### Experimental setup

This was the same as in the pilot study, except for the SCR measurement. The SCR remote transmitter with a strap was worn on the participant's left wrist. The SCR data were recorded with a Biopac MP100 acquisition unit and AcqKnowledge software.

#### Design

There were two within-group factors: First, the movement of the virtual hand and the vibration of the sensors were either synchronous or asynchronous with the movement of the actual hand and the event-hand contact. Second, the virtual hand was either contacted by a ball or cut by a knife, in both cases accompanied by tactile vibration. The order of the two synchronicity conditions was balanced across participants, as was the order of the two types of events.

#### Procedure

The procedure was very similar to that in the pilot study. Participants wore the data glove on their right hand and the orientation tracker on their right wrists, and the SCR finger electrodes on their left hand. They were to put their real right hand into a black box placed between them and the computer screen (Figure 1B). Participants would then see a virtual ball or knife (depending on the trial) coming down slowly from the top of the screen to approach and eventually touch the palm of their virtual hand. The ball would bounce several times, every time accompanied by the vibration, just like in the pilot study. The knife would approach and eventually cut the palm of the virtual hand, accompanied by the vibration, and some blood would appear from the cut. Both events were shown under synchronous and asynchronous conditions. All remaining details were as in the pilot study, and the same questionnaire was used.

#### SCR measurements

Following previous studies (e.g., Armel and Ramachandran, 2003) we used SCR to assess affective reactivity (see Figner and Murphy, 2010; Boucsein, 2011). SCR is a standard physiological measure and the best predictor of psychological arousal, and the fact that participants cannot control their SCR voluntarily makes it a reliable measure. We measured SCR during the entire experiment. We defined a latency onset window between 1 and 5 s after stimulus/event onset, with the skin conductivity before stimulus/event onset serving as baseline. We then calculated the magnitude of the event-induced SCR by subtracting baseline skin conductivity from the peak amplitude of the SCR during the analyzed time window, and took the log(magnitude+1).

### Results

#### Questionnaire

**Figure 3:**
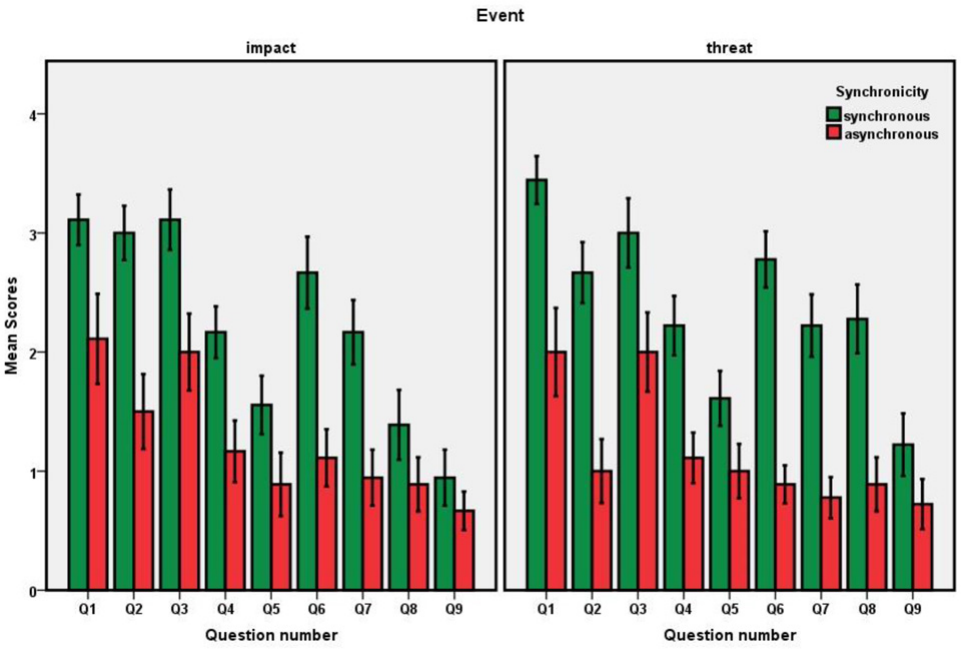
**Experiment: Boxplots for the questionnaire scores as a function of event type (impact vs. threat) and synchronicity**.

We analyzed the responses to all 9 items by means of a 2 (synchronicity) × 2 (event type) ANOVA. For Questions 1–7 the analyses yielded reliable main effects of synchronicity (see Figure 3 and Table 2 for *p*-values) but no other effects, except Questions 8 and 9, all event *p*s > 0.06. Hence, all critical questions provided evidence for a VHI, and the illusion did not depend on event type.

**Table 2:** **Experiment: Means and standard deviations (in brackets) for the questionnaire scores**.

	**Q1**	**Q2**	**Q3**	**Q4**	**Q5**	**Q6**	**Q7**	**Q8**	**Q9**
Impact synchronous	3.11 (0.900)	3.00 (0.970)	3.11 (1.079)	2.17 (0.924)	1.56 (1.042)	2.67 (1.283)	2.17 (1.150)	1.39 (1.243)	0.94 (0.998)
Impact asynchronous	2.11 (1.605)	1.50 (1.339)	2.00 (1.372)	1.17 (1.098)	0.89 (1.132)	1.11 (1.023)	0.94 (0.998)	0.89 (0.963)	0.67 (0.686)
Threat synchronous	3.44 (0.856)	2.67 (1.085)	3.00 (1.237)	2.22 (1.060)	1.61 (0.979)	2.78 (1.003)	2.22 (1.114)	2.28 (1.227)	1.22 (1.114)
Threat asynchronous	2.00 (1.572)	1.00 (1.138)	2.00 (1.414)	1.11 (0.900)	1.00 (0.970)	0.89 (0.676)	0.78 (0.732)	0.89 (0.963)	0.72 (0.895)
*p* (synchronicity)	0.002	<0.001	0.002	<0.001	<0.001	<0.001	<0.001	0.002	0.135
*p* (event)	0.659	0.069	0.726	1.000	0.692	0.762	0.772	0.042	0.010
*p* (interaction)	0.163	0.564	0.777	0.726	0.886	0.302	0.495	0.011	0.298

P-values for the main effects of synchronicity are shown in the last row.

#### SCR

**Figure 4:**
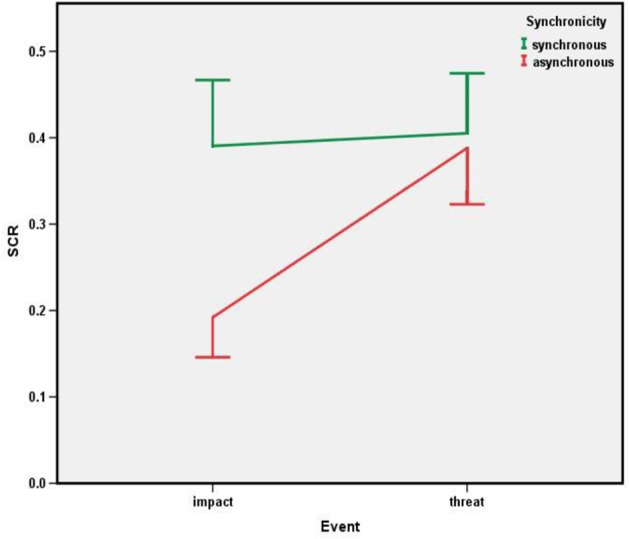
**Experiment: SCR as a function of event type (impact vs. threat) and synchronicity**.

We analyzed the event-induced SCRs by means of a 2 (synchronicity) × 2 (event type) ANOVA and used two-tailed *t*-tests for more detailed analyses. Figure 4 shows the outcome. The main effect of synchronicity was reliable, *F*_(1, 17)_ = 6.046, *p* = 0.025, as was the type of event effect, *F*_(1, 17)_ = 4.601, *p* = 0.047. However, both effects were mediated by a reliable interaction, *F*_(1, 17)_ = 5.069, *p* = 0.038. Figure 4 shows that synchronicity clearly mediates SCR in the impact condition, producing a reliably higher peak in SCR in the synchronous as compared to the asynchronous condition, *p* = 0.010. This is no longer the case in the threat condition, where peaks are high in both synchronicity conditions and no longer statistically different, *p* = 0.732.

## General discussion

The aim of this study was to compare the effect of perceived events that either impact or threaten a virtual hand on perceived ownership and affective resonance. The findings from the impact condition fully replicate the observations of Yuan and Steed (2010). Even though we compared synchronous and asynchronous conditions (with the same effector) while Yuan and Steed compared a synchronous virtual hand with a synchronous virtual arrow, the outcomes are comparable: conditions that induce perceived hand ownership are associated with greater affective responses if the “owned” virtual hand becomes the target of some sort of impact. At the same time, the findings from our threat condition show that this interaction between ownership and affective resonance does not generalize to events that are more serious. This amounts to a partial non-replication of Yuan and Steed (2010) and suggests that their manipulation actually represented “impact” targeting the virtual hand rather than real “threat.”

The outcome of the threat condition, in turn, is consistent with previous neuroscientific observations, suggesting that threat targeting another person produces affective responses comparable to those people show when being a target of threatening events themselves (Morrison et al., 2004; Singer et al., 2004). As our findings demonstrate, such evidence of affective resonance is not restricted to body parts that belong to other people but can also be observed for unconnected body parts that are not owned by, or associated with a person or agent. Interestingly, the degree of affective resonance was statistically independent of ownership, suggesting that people care much less about who is being attacked if a threat is only sufficiently serious. It is thus possible that cross-personal affective resonance can only be obtained in the face of events that are as serious as a flesh-cutting knife or a pricking needle (as in several neuroimaging studies), but not with less damaging events. In other words, people's emotional involvement in the fate of others may be restricted to really dangerous incidents.

One possible take of these observations would be that people are more likely to neglect the actual ownership of body parts in the face of threat. This could be because threats (but not mere impact) induce stress-like states that are accompanied by an excessive turnover of dopamine in the prefrontal cortex and other areas, which again is known to result in cognitive impairments (e.g., Deutch and Roth, 1990; Murphy et al., 1996). However, such a threat-induced relaxation of ownership criteria should have affected the ownership-related judgments obtained in the questionnaire, which however did not show any impact of event type. This renders a stress- or ownership-neglect approach of our observations less attractive.

Another take relates to the claim that affective responses might be triggered along two different neural pathways (LeDoux, 1998): a slow, cortical pathway that processes all the available information and computes the emotional relevance based on the available knowledge and past experience and a fast, subcortical pathway mediated by the amygdala, which can directly access action and arousal systems, and hijack the cognitive apparatus in the face of threat. It makes sense to assume that our knife condition was sufficiently threatening to activate the fast, direct pathway while the ball condition did not. As a consequence, the emotional reactions in the ball condition were mediated by cognitive processes including considerations about hand ownership, which triggered affective responses in a top-down fashion in the synchronous condition only. In contrast, the emotional reactions in the knife condition might have been triggered more in a bottom-up fashion, thereby shortcutting ownership considerations. We speculate that a similar scenario holds for the successful neuroscientific demonstrations of affective resonance to the fate of “non-owned” body parts belonging to other individuals. In any case, however, our findings strongly suggest that perceived body ownership and affective responses to body-related impact or threat can be dissociated and are thus unlikely to represent the same underlying process.

### Conflict of interest statement

The authors declare that the research was conducted in the absence of any commercial or financial relationships that could be construed as a potential conflict of interest.
